# Use of Motion Management to Model Unmanaged Motion: Dosimetric Consequences of Unmanaged Prostate Motion Assessed Using Real-Time Radiotherapy Adaptation Data

**DOI:** 10.7759/cureus.82620

**Published:** 2025-04-20

**Authors:** Scott B Crowe, Jemma Walsh, Gregory Rattray, Philip Chan, Tanya Kairn

**Affiliations:** 1 Cancer Care Services, Royal Brisbane and Women's Hospital, Brisbane, AUS; 2 School of Chemistry and Physics, Queensland University of Technology, Brisbane, AUS; 3 School of Electrical Engineering and Computer Science, University of Queensland, Brisbane, AUS

**Keywords:** motion management, prostate cancer, radiotherapy, real-time tracking, tomotherapy

## Abstract

This case report presents a novel application of intrafraction prostate motion data from the Radixact Synchrony real-time radiotherapy treatment adaptation system (Accuray Inc., Sunnyvale, USA) to evaluate the dosimetric consequences if a specific patient treatment had been delivered without the use of real-time adaptation, i.e., if the prostate motion had been unmanaged.

The patient included in this study was selected from a dataset of 50 completed prostate Radixact Synchrony treatments for which intrafraction motion had been collected. This patient was identified as having the highest proportion of fractions where the target motion exhibited continuous drift, a form of motion that is particularly likely to degrade treatment accuracy, especially when combined with the continuous superior motion of the Radixact treatment couch.

Target trajectory data from the Synchrony real-time adaptation system were collected for this patient using the Delivery Analysis software (Accuray Inc.) and used, via a novel application of a deformation vector field that took couch motion interplay into account, to perform several voxel-wise deformations of the patient’s planned treatment dose distribution to cover various intrafraction motion scenarios for two treatment regimens.

For the standard hypofractionated prostate radiotherapy treatment regimen used in this patient's clinical treatment, 60 Gy in 20 fractions, this study showed that target coverage would have been compromised for this patient had the Synchrony system not been used to adapt the radiation beam to account for the observed prostate motions. Evidently, this patient benefited from the use of real-time target tracking even though an ultra-hypofractionated stereotactic body radiotherapy (SBRT) regimen was not used in this case.

By rescaling the patient's hypofractionated dose distribution to model an ultra-hypofractionated SBRT treatment regimen, this study also suggests that a substantial and clinically unacceptable target underdosage might have occurred if this patient had been treated with SBRT and 3 mm margins without the use of the Synchrony system. This result points to an even greater benefit from Synchrony real-time adaptation for SBRT prostate treatments.

## Introduction

The unmanaged motion of a tumor or target volume during radiotherapy treatments has the potential to compromise prescription dose coverage and result in poor treatment outcomes. For prostate cancer, intrafraction motion during treatment delivery may be irregular and unpredictable in nature due to, for example, peristalsis in surrounding organs. Maximum prostate displacements from 5 mm to approximately 20 mm have been reported in the literature [1-5].

Intrafraction target motion has historically been managed via the use of target expansion margins encompassing the uncertainty in target position and possible range of motion, though this approach inevitably increases the amount of normal or critical tissues irradiated. Real-time adaptation techniques, including gating and motion synchronization, have avoided this by monitoring target position with on-board or in-room imaging systems during treatment. Real-time monitoring and adaptation have been reported to be effective for management of intrafraction motion of the prostate when tight margins are required or for long treatments [6-7].

The Accuray Radixact platform, a helical tomotherapy treatment system, is packaged with the Synchrony real-time adaptation solution (Accuray Inc., Sunnyvale, USA). When treating pelvic or abdominal tumors with the Synchrony system, radiographic images are frequently acquired to monitor the position of implanted fiducials, and the position of the Radixact’s jaws and multi-leaf collimator are continuously adjusted to achieve accurate treatment targeting. The use of this system in the treatment of prostate cancer with a stereotactic body radiotherapy regimen has been described previously by Shintani et al. [8]. An additional benefit of the Synchrony real-time adaptation system is that it facilitates the collection and analysis of target trajectory data via the Delivery Analysis software (Accuray Inc.) [9].

Since commissioning the Radixact Synchrony platform in 2023, our department has treated more than 80 prostate cancer patients with Synchrony fiducial tracking. The commissioning and subsequent ongoing quality assurance of the system have been consistent with the recommendations of the American Association of Physicists in Medicine (AAPM) Task Group Report 306 [10], including end-to-end testing with moving phantoms. Our experiences with validating the accuracy of motion adaptation with a robotic arm phantom have been described by Hindmarsh et al. [11].

This case report explores the dosimetric benefit of motion synchronization in helical tomotherapy for a single patient with frequent continuous intrafraction drift of the prostate. Target trajectory data from the Synchrony real-time adaptation system were collected for this patient using the Delivery Analysis software and used, via a novel application of a deformation vector field, to characterize the dosimetric consequences that would have occurred if the patient had been treated without the use of Synchrony, i.e., if the prostate motion had been unmanaged. This case report thereby evaluates the potential benefit of real-time adaptation for this patient in various clinical scenarios, including the use of an ultra-hypofractionated regimen.

## Case presentation

Patient and prescription

The patient included in this study was selected from a dataset of 50 completed prostate Radixact Synchrony treatments for which intrafraction motion had been characterized as the patient with the highest proportion of fractions where the target motion exhibited sustained continuous drift exceeding 3 mm in magnitude. The AAPM Task Group Report 264 [12] noted that these drifts, or baseline shifts, may be of significant concern, whereas small periodic motions may have a negligible effect on overall treatment accuracy. Tudor et al. [13] reported that for helical radiotherapy, even in a treatment that was subject to periodic respiratory motion, interplay between the motion of jaws across the target and baseline variations in tumor position were observed to be a greater contributor to dose variation.

The patient selected for this case study was a 78-year-old male patient who presented with adenocarcinoma of the prostate with staging T2c N0 M0, a Gleason score of 4+3, pre-treatment prostate-specific antigen (PSA) of <6, and overall stage IIc. Three months prior to treatment, three gold seed fiducials were surgically implanted under trans-rectal ultrasound guidance. At the time of radiation therapy, the patient was undergoing androgen deprivation therapy and no chemotherapy. Bladder preparation included emptying his bladder and then drinking 400 ml of water 30 minutes prior to CT simulation and treatment fractions. The patient was encouraged to maintain a regular bowel habit and advised to stay hydrated and take macrogol if required to ensure bowel motion within two hours before CT simulation and treatment. Loperamide was provided during treatment for diarrhea relief.

**Figure 1:**
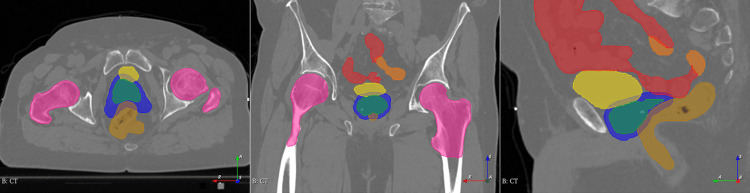
Radiation therapy simulation CT and planning contours The CT image of the patient includes contours for clinical target volume (green), planning target volume (blue), bladder (yellow), rectum (brown), bowel (red), sigmoid colon (orange), and femoral heads (pink).

The patient's simulation CT image was fused with their MRI using fiducial seed matching, and the fused MRI data was used to contour a clinical target volume (CTV) containing the prostate and seminal vesicles, which had a total volume of 43.3 cm³. The simulation CT was used to contour the rectum from the sigmoid junction to the bottom of the ischial tuberosities or anal verge. The simulation CT image and contours are shown in Figure 1.

The patient treatment was prescribed and planned according to Australian consensus guidelines published in the eviQ protocol for definitive hypofractionated external beam radiotherapy of prostate adenocarcinoma (ID 3370 version 3, since replaced with ID 234 version 7) [14, 15]. This included a prescription of 60 Gy in 20 fractions to the prostate and proximal seminal vesicles, with a planning target volume (PTV) defined by expanding the CTV using a 6 mm margin. This consensus protocol defines acceptable dose-sparing constraints for the rectum, bladder, and femoral heads [15]. All dose-volume objectives were satisfied by the planned treatment dose distribution.

Treatment planning

The treatment was planned with the Accuray Precision treatment planning system (version 3.3.1.0), using a total of 17 optimization objectives. Treatment parameters included a field width of 2.5 cm and a pitch of 0.303. The planned treatment had a delivery time of 187.3 seconds, with a gantry rotation time of 18.8 seconds. The optimized dose distribution satisfied the 13 dose objectives defined in the eviQ protocol [15] for the rectum, bladder, and femoral heads while maintaining a median dose of 60.1 Gy to the planning target volume.

Six planar radiographic images were planned for each gantry rotation, resulting in 60 planned images for each fraction (which could be exceeded when there was a pause in treatment). The associated imaging dose was approximately 0.9 cGy per fraction. The placement of fiducials and the imaging angles were validated by visual inspection of the digitally reconstructed radiographs by the treatment planner and with the score wheel tool. Synchrony treatment parameters included an auto-pause delay of 20 seconds, a tracking range of 20 mm, medium sensitivity, and use of the XL pelvis imaging protocol.

Real-time adaptation data

**Figure 2:**
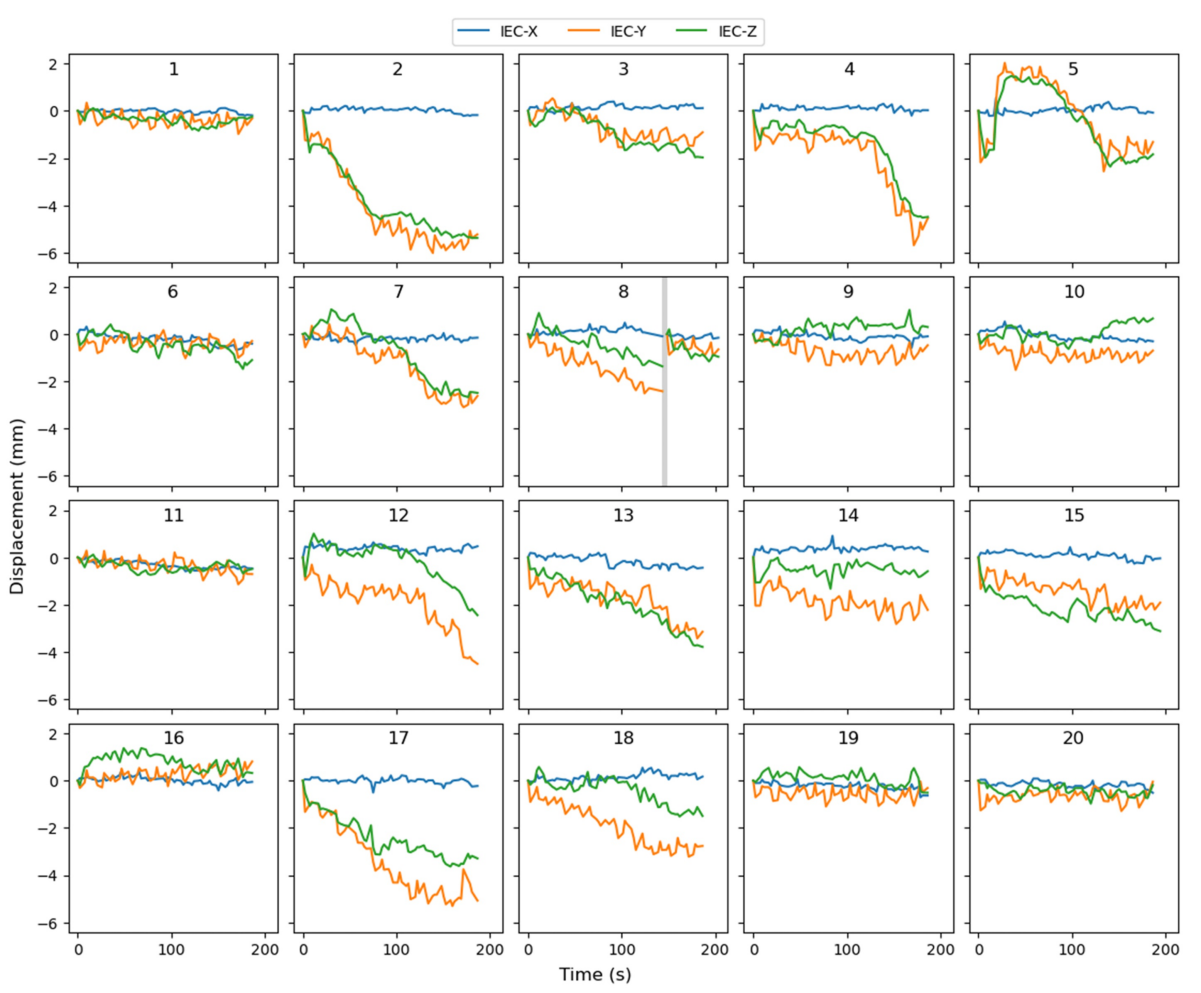
Motion of prostate fiducials during fractional treatment deliveries The IEC-X (left-right, blue), IEC-Y (superior-inferior, orange), and IEC-Z (anterior-posterior, green) motion for all 20 fractions. Graphs are labelled with fraction numbers from fraction 1 (top left) to fraction 20 (bottom right). There was a pause in treatment delivery in fraction 8, represented by a vertical grey bar.

Following completion of the patient's treatment, motion data describing displacement of fiducials from their initial treatment positions were extracted from the Accuray Delivery Analysis software. Figure 2 shows the prostate motion across the 20 fractions, derived from Delivery Analysis data and plotted using an in-house Python code [9].

Worst-case continuous drift motion can be seen in fraction 2, shown second from the left in the top row of Figure 2. During the delivery of this fraction, the prostate gradually moved nearly 6 mm in the inferior and posterior directions.

For this patient, all prostate motions indicated by the data in Figure 2 were corrected by the Radixact Synchrony system through real-time adjustments of the jaws and multi-leaf collimator leaves, though the precision of adaptation for left-right and anterior-posterior motion is limited to approximately 3.1 mm, given the width of the Radixact system's multi-leaf collimator leaves.

Deformation vector field transformation

To assess the potential dosimetric impact of unmanaged motion in these fractions, voxel-wise translation by deformation vector fields was used [16]. In this context, a deformation vector was the three-dimensional displacement where the dose was deposited in the patient due to unmanaged motion, and the voxel-wise deformation vector field was the definition of displacement for each dose value, or voxel, in the treatment planning system calculation. Deformation vector fields have been used for dose deformation and summation when accounting for interfraction motion [16]. Other strategies for modeling the impact of tumor motion, including recalculation of dose with isocenter shifts, the use of synthetic four-dimensional CT data incorporating simulated organ motion, convolution of the static dose cloud and distribution of target motion [2], and simple affine transformations were considered; however, these approaches do not account for potential correlation or interplay between couch and tumor motion.

Because the Radixact couch moves in the superior direction during treatment delivery, the dosimetric impact of continuous unmanaged drift of the target volume in the inferior direction is equivalent to a compression of the dose to the target along the longitudinal axis. That is, it results in an increased dose in the superior portion of the target (as the target has moved in the same direction as the field) and a potential lack of coverage of the target, or undershoot, at the inferior edge of the target. Conversely, superior motion of the target will result in a decreased dose in the target and overshoot at the inferior edge. This effect cannot be easily modeled with established dose convolution approaches. Accurate dose recalculations would need to account for both tumor displacement and other anatomical changes at each control point.

Accordingly, to account for the specific movements detected by the Synchrony system for this patient while accounting for the continuous superior motion of the treatment couch, a novel application of the deformation vector field approach was developed. For all voxels within each 1-mm-thick transverse slice of the precision-calculated dose distribution, the voxel dose translation vector was defined as the reverse of the target motion vector for the couch position corresponding to the transverse dose slice.

Specifically, for all transverse slices corresponding to a couch position used during treatment, the deformation vector was interpolated from the observed motion using a time corresponding to the fraction of couch travel that occurred. For transverse dose planes superior to the starting couch position, it was assumed that no deformation occurred, that is, there is no target motion at the start of treatment. For transverse planes inferior to the final couch position, the target motion vector at the end of treatment was used.

**Figure 3:**
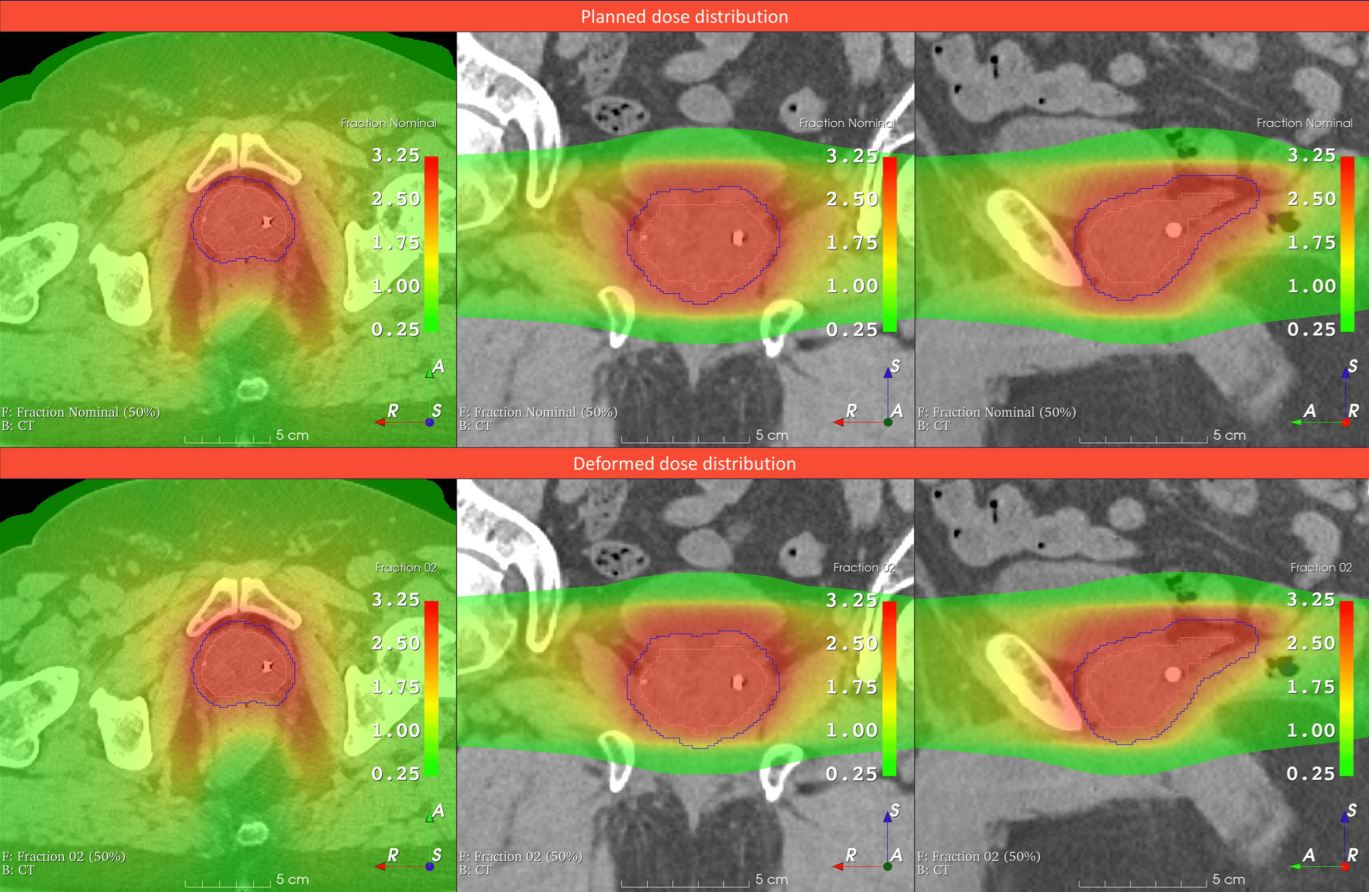
Planned and voxel-wise deformed dose distributions The voxel-wise dose deformation for fraction 2 has been applied to the nominal fractional dose (top) to produce the deformed dose distribution (bottom). The deformation has resulted in the dose being shifted superiorly and anteriorly due to the drift of the target in the inferior and posterior directions.

The resulting deformation vector field array was applied to the precision-calculated dose distribution by using the SimpleITK DisplacementFieldTransform function [17] within our in-house Python code. The resulting dose distributions were analyzed using 3D Slicer software [18]. An example of a transformed dose distribution for the worst-case continual drift seen during fraction 2 delivery (see Figure 2) is shown in Figure 3.

Unmanaged motion scenario: standard hypofractionated treatment

**Figure 4:**
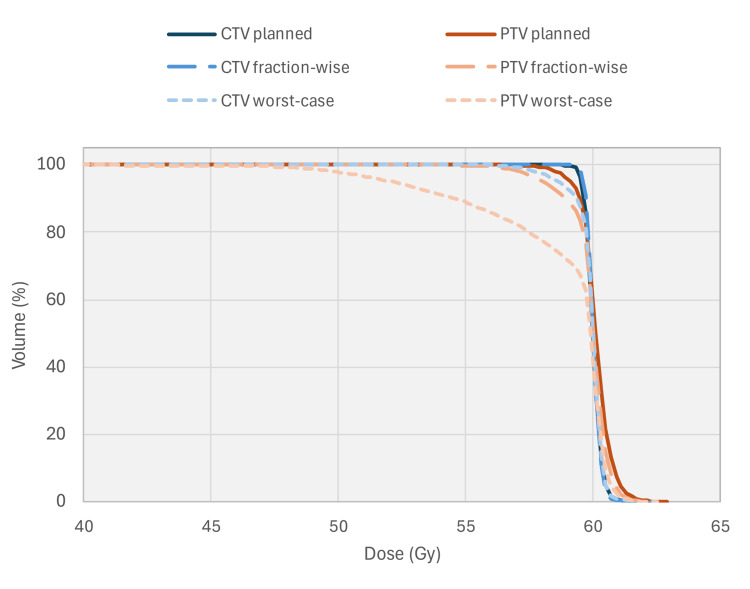
CTV and PTV dose-volume histograms for planned, fraction-wise, and worst-case deformation scenarios for standard hypofractionated treatment. The dose axis is shown starting at 40 Gy to allow improved delineation of histogram lines. CTV: clinical target volume; PTV: planned target volume

The dose was deformed for two scenarios: fraction-wise, where the fractional dose was deformed using deformation vector fields generated from respective fractional motion, and all resulting fractional doses were summed; and worst-case, where the total dose was deformed using the deformation vector field generated with the motion observed during fraction 2, which had the largest continuous drift, and the resulting single-fraction dose was multiplied by the total number of fractions in the treatment. Resulting dose volume histograms for the CTV and PTV are shown in Figure 4.

The difference in D95% was +0.1 Gy and -0.8 Gy in the CTV and -0.5 Gy and -6.9 Gy in the PTV for the fraction-wise and worst-case scenarios, respectively. This suggests that, for this patient, with a CTV to PTV expansion of 6 mm, the CTV could have received a dose consistent with the prescription without Synchrony's motion management only if all other aspects of the treatment were delivered without uncertainty.

Unmanaged motion scenario: ultra-hypofractionated treatment

To assess the potential difference for treatments where a smaller margin is used, a new mock CTV was created for the existing treatment by subtracting 3 mm from the PTV. A minimum CTV-to-PTV margin of 3 mm is suggested by the Australian consensus protocol, eviQ, for definitive ultra-hypofractionated stereotactic external beam radiotherapy of prostate adenocarcinoma (ID 4140) [19]. This stereotactic ablative body radiotherapy (SBRT) protocol includes a prescription of 40 Gy delivered in 5 fractions. To mimic the use of this protocol, the total and fractional dose distributions were renormalized to 40 Gy and 8 Gy, respectively.

**Figure 5:**
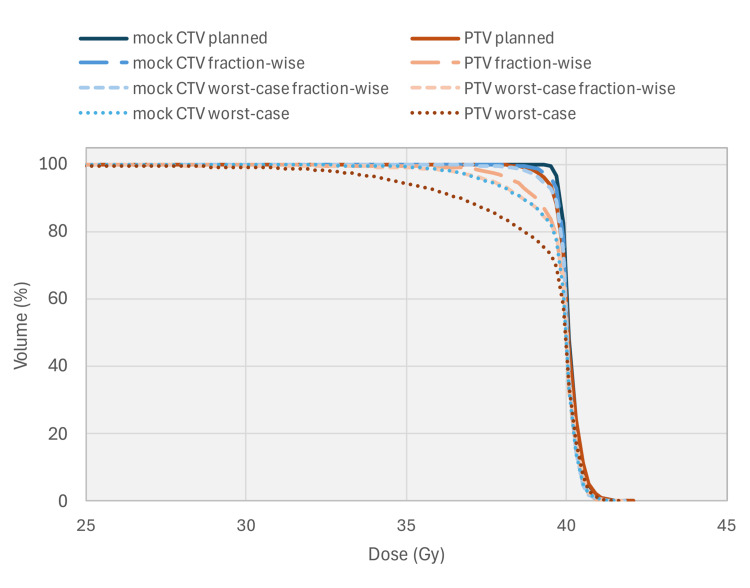
Mock CTV and PTV dose-volume histograms for planned, fraction-wise, worst-case fraction-wise, and worst-case convolution scenarios for simulated ultra-fractionated treatment. The dose axis is shown starting at 25 Gy to allow improved delineation of histogram lines. CTV: clinical target volume; PTV: planned target volume

The renormalized dose distributions were deformed for three scenarios: fraction-wise, where the fractional dose was deformed using deformation vector fields generated from the first five fractions of treatment; worst-case fraction-wise, where the deformation vector fields were generated from the five fractions of the treatment with the largest motion (fractions 2, 4, 12, 13, and 17; see Figure 2); and worst-case, where the deformation vector fields were generated from the motion observed during fraction 2 (Figure 2). Dose volume histograms for the CTV and PTV are shown in Figure 5 for these simulated ultra-hypofractionated treatments.

The differences in D95% were -0.2 Gy, -0.4 Gy, and -2.1 Gy in the mock CTV and -1.0 Gy, -1.7 Gy, and -4.6 Gy in the PTV for the fraction-wise, worst-case fraction-wise, and worst-case scenarios, respectively. For the worst-case scenario, the 2.1 Gy difference in D95% corresponds to 5.4% of the prescribed dose. The mock CTV volume covered by 95% of the prescription, V95%, decreased from 100% coverage in the nominal dose distribution to 93.3% coverage for the worst-case scenario, which exceeds clinically acceptable limits and represents a risk of loss of local tumor control. These results suggest that a substantial and clinically unacceptable target underdosage could have occurred if this patient had been treated with SBRT and 3 mm margins and exhibited the worst-case motion without the use of the Synchrony system.

## Discussion

Figure 2 provides an example of the detailed target motion information that can be extracted from Synchrony Delivery Analysis data. For the treatment examined in this case report, the data in Figure 2 indicate minimal left-right motion; however, this patient was selected for use in this case report due to substantial superior and posterior continuous drifts being detected in several treatment fractions. During this patient's treatment, these intrafraction motions were generally corrected in real-time by the Radixact Synchrony system, with the exception of residual left-right and anterior-posterior motions smaller than half the width of the Radixact multileaf collimator leaves (i.e., smaller than 3.125 mm). Nonetheless, the manipulation of the planned treatment dose distribution using a voxel-wise vector deformation field derived from the intrafraction prostate motions detected by the Synchrony system allowed the consequences of delivering this treatment without real-time adaptation to be evaluated.

A limitation of the voxel-wise transformation approach is that it treats the dose distribution as a static cloud that can be deformed without being affected by the surrounding anatomy. For example, this method does not take into account changes in radiological path lengths due to the prostate changing its location relative to static bony anatomy.

Ideally, the Accuray Precision treatment planning system would be used to achieve dose calculations that include intrafraction anatomical changes, where some, but not all, tissue has gradually moved during treatment delivery. However, the accurate calculation of dose incorporating this complex motion and interplay with couch translation would require individual calculations for many control points or treatment fragments, each with an associated CT dataset capturing target motion. While such highly complex calculations remain beyond the scope of commercial treatment planning systems, the vector deformation field method used for this case report may be regarded as an acceptable surrogate for the ideal four-dimensional dose calculation that would be needed to fully illustrate these dosimetric effects.

Figure 4 indicates the dosimetric consequences if this patient's 60 Gy in 20 fractions (hypofractionated) treatment had been delivered as planned, except without real-time adaptation. With a CTV to PTV expansion of 6 mm, and with all detected motions being within 6 mm, this result suggests that even when couch motion interplay is taken into account via the deformation vector field method, the CTV prescription would have been met even in the worst-case scenario. However, this observation accommodates no other margin for error, wholly relying on the assumption that no other geometric uncertainties contribute to PTV margin selection. Given that PTVs generally account for a range of treatment uncertainties (from simulation to target contouring to treatment delivery system performance), reliance on the 6 mm PTV margin to ensure CTV coverage would have been inadvisable without real-time adaptation or some other form of motion management if the worst-case motion described in this case report occurred.

Figure 5 shows the dosimetric consequences if the patient had been treated with an ultra-hypofractionated regimen, 40 Gy in 5 fractions with a CTV-PTV expansion margin of 3 mm, again without real-time adaptation. In this case, there is a clear loss of CTV coverage and loss of expected tumor control if real-time adaptation is not used, especially in the worst-case scenarios where the fractions with the largest continuous drift motions were used to calculate the dose deformation vector fields.

The use of the mock CTV to assess the potential impact of unmanaged prostate motion for ultra-hypofractionated SBRT prostate treatments relies on two assumptions. The first assumption was that the renormalized dose distribution included in this study is broadly representative of the dose distribution that would have been produced if the treatment had been originally planned as SBRT. This may not be the case given differences in target definition (e.g., inclusion of seminal vesicles), differences between dose prescription to the CTV and PTV to allow non-homogeneous dose, and differences in critical organ-at-risk dose-sparing constraints for SBRT treatments. In this study, the renormalized dose did not satisfy the ultra-hypofractionated organ-at-risk dose-sparing constraints recommended in the eviQ protocol [19], and yet this dose distribution has been used to exemplify the effects of intrafraction motion on target dose coverage. Changes in prescription and dose optimization for SBRT could also require changes in pitch, gantry rotation speed, and jaw size, which could impact real-time tracking and adaptation.

The second assumption was that the intrafraction motion of the prostate for an ultra-hypofractionated SBRT prostate treatment and the treatment included in the study are similar. Higher doses per fraction would result in increased delivery times, which may mean increased displacement. There would be obvious anatomical limits to these displacements. I.e., anatomy that moved 10 mm during a 10-minute treatment delivery cannot move 20 mm during a 20-minute treatment delivery if surrounding tissues or nearby bony anatomy physically prevent such large motions. The motion trajectories identified for the patient in this study, shown in Figure 2, generally plateau at or before 6 mm, even when treatment durations appear to allow further motion to occur (for example, in fractions 2, 4, and 17 in Figure 2). This feature suggests that the anatomical limitations on this motion had already been reached for this patient during each of the 3 Gy fractions of the treatment and that more extreme motions would be unlikely even if the dose per fraction was increased to 8 Gy, as in the ultra-hypofractionated scenario.

Despite the limitations noted above, the results of this study clearly indicate that where real-time adaptation solutions such as Radixact Synchrony are not available, care must be taken when selecting CTV-PTV expansion margins. Narrow planning target volume margins for prostate radiotherapy have been previously associated with reduced rates of freedom from biochemical failure when relying only on image guidance during patient positioning [20].

## Conclusions

In this case report, data derived from using the Radixact Synchrony system for real-time tracking and accurate irradiation of a prostate radiotherapy target have been used to deform the patient's planned dose distribution to account for the target motions and couch interplay that would have occurred if the patient had been treated without real-time adaptation. Through a novel application of a deformation vector field that took couch motion interplay into account, it was shown that in the worst-case scenario, target coverage would have been compromised for this patient had the Synchrony system not been used to direct the radiation beam to account for the observed prostate motions. Evidently, this patient benefited from the use of real-time target tracking even with the use of a standard 60 Gy in 20-fraction hypofractionated treatment regimen and a 6 mm CTV-PTV expansion margin.

By rescaling the patient's hypofractionated dose distribution to model an ultra-hypofractionated SBRT treatment regimen, the results of this study also point to a substantial and clinically unacceptable target underdosage, which might have occurred if this patient had been treated with SBRT and 3 mm margins without the use of the Synchrony system. The method of simply rescaling a non-SBRT dose distribution to model an SBRT treatment, without taking changes in organ-at-risk tolerances and plan optimization objectives into account, clearly has limitations, and so this aspect of the case report should be validated using clinical SBRT treatment plans in the future. Nonetheless, the method of generating voxel-wise dose deformation vector fields, informed by real-time tracking of radiotherapy treatment targets, has immediate value for informing radiotherapy margin selection as well as substantial potential for guiding the development of the radiotherapy treatment planning and delivery techniques of the future.
